# Scotch Medical Colleges

**Published:** 1846-12

**Authors:** 


*Scotch Medical Colleges.*—The University of St. Andrew's is the only one of these colleges which grants degrees to candidates who do not reside in the towns in which they are situated for at least one session, and take out certificates from their professors. Fellows, licentiates, and members of the colleges of surgeons, are admitted to examination on production of their diplomas: and all other candidates are required to produce the usual certificates obtained during four complete winter sessions. The periods of graduation are the first Tuesday in May and the first Tuesday in August. As many surgeons throughout England and Ireland are desirous of obtaining the title of Doctor of Medicine without absenting themselves from home for any length of time, and many students are anxious to secure the same qualification without being obliged to pay for additional certificates, it is desirable that these regulations should be generally known. The only two institutions which grant degrees without residence, or payment of extra fees to particular professors, are the Universities of London and St. Andrews. They both, however, require as protracted and perfect a course of study, and submit the candidate to as severe examinations, as any of the other colleges. Formerly the University of St. Andrew's granted its diploma to medical practitioners without examination, by which that qualification was brought into discredit; but for some years it has been even more strict than many other establishments for medical education. In our Student's Number of last year we printed the regulations of St. Andrew's University in full, with a detailed account of the examination, which may be obtained at the office of this journal.

The Universities of Edinburgh and Glasgow require four years' study and a year's residence, with the usual certificates. That of Glasgow receives certificates from all schools; but that of Edinburgh has, until lately, preferred those obtained at medical colleges. In this respect it appears it has at length given way, having seen the necessity of keeping pace wtih the progress of events, and submitting to the test of competition.

The degrees granted by the Scotch colleges cost about £25.—*Dub. Med. Press.*

